# Fast multiclonal clusterization of V(D)J recombinations from high-throughput sequencing

**DOI:** 10.1186/1471-2164-15-409

**Published:** 2014-05-28

**Authors:** Mathieu Giraud, Mikaël Salson, Marc Duez, Céline Villenet, Sabine Quief, Aurélie Caillault, Nathalie Grardel, Christophe Roumier, Claude Preudhomme, Martin Figeac

**Affiliations:** Laboratoire d’Informatique Fondamentale de Lille (LIFL, UMR CNRS 8022, Université Lille 1) and Inria Lille – Cité scientifique – Bâtiment M3, 59655 Villeneuve d’Ascq, France; Functional and Structural Genomic Platform, Université Lille 2, IFR 114 Lille, France; Department of Hematology, Biology and Pathology Center, Lille, France; Inserm U-837, Cancer Research Institute, Lille, France; Lille Institute for Cancer Research (IRCL), Lille, France; SIRIC OncoLille, Lille, France

**Keywords:** Sequence analysis, High-throughput sequencing, V(D)J recombinations, Repertoire sequencing, Immunology, Leukemia, Minimal residual disease follow-up

## Abstract

**Background:**

V(D)J recombinations in lymphocytes are essential for immunological diversity. They are also useful markers of pathologies. In leukemia, they are used to quantify the minimal residual disease during patient follow-up. However, the full breadth of lymphocyte diversity is not fully understood.

**Results:**

We propose new algorithms that process high-throughput sequencing (HTS) data to extract unnamed V(D)J junctions and gather them into clones for quantification. This analysis is based on a seed heuristic and is fast and scalable because in the first phase, no alignment is performed with germline database sequences. The algorithms were applied to TR *γ* HTS data from a patient with acute lymphoblastic leukemia, and also on data simulating hypermutations. Our methods identified the main clone, as well as additional clones that were not identified with standard protocols.

**Conclusions:**

The proposed algorithms provide new insight into the analysis of high-throughput sequencing data for leukemia, and also to the quantitative assessment of any immunological profile. The methods described here are implemented in a C++ open-source program called Vidjil.

**Electronic supplementary material:**

The online version of this article (doi:10.1186/1471-2164-15-409) contains supplementary material, which is available to authorized users.

## Background

*V(D)J recombinations.* V(D)J recombinations in lymphocytes are essential for immunological diversity because they influence the production of antibodies and antigen receptors [1, 2]. VDJ recombinations occur in B-cell heavy chains (IgH) and T-cell *β* and *δ* chains (TR *β* and *δ*), whereas VJ recombinations occur in B-cell light chains *κ* (Ig *κ*) and *λ* (Ig *λ*), and T-cell *α* and *γ* chains (TR *α* and *γ*).

**Figure 1:**
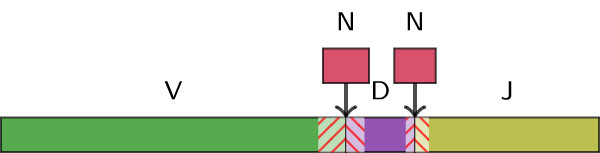
**An V(D)J recombination in a lymphocyte derives from two (or three) germline V, (D), and J genes that may have been truncated or mutated.** The N-diversity regions correspond to random nucleotides inserted between the rearranged genes. Typical V genes are between 250 and 310 bp, D genes between 10 and 35 bp, and J genes between 40 and 70 bp.

The total repertoire of immunoglobulin (Ig) and T-cell receptor (TR) molecules is estimated to include nearly 10^12^ molecules, resulting from combinatorics of V(D)J recombinations, somatic mutations, deletions at junction sites, and the addition of N-diversity regions between the rearranged genes [3] (see Figure 1). A study found at least one million recombinations among the T cells in a single blood sample from one patient [4].

*Acute lymphoblastic leukemia (ALL).* Acute lymphoblastic leukemia is a lymphoid malignancy mainly affecting children. In more than 90% of cases, a recombined Ig or TR junction fingerprint of the blastic cells can be identified easily at diagnosis. This clonality marker is used during patient follow-up to quantify the minimal residual disease [3, 5].

The survival rate of ALL patients has improved in recent decades thanks to its accurate diagnosis and better therapeutic stratification according to prognostic factors. These prognostic factors can be determined at the time of diagnosis, but also throughout the follow-up period when the minimal residual disease is monitored after therapy. Monitoring requires the analysis of both lymphoid cells (lymphoblasts) and normal lymphocytes in the peripheral blood, and these cells are counted according to their V(D)J recombinations. For better follow-up efficacy, clonal recombinations must be detected at lower concentrations than are possible with current techniques (Biomed-2 and qRT-PCR [3], or flow cytometry [6]). More importantly, current techniques are not adapted to follow populations of various clones [7]. Consequently, they are unable to detect a relapse attributable to a clone other than the one identified at diagnosis.

*Software for V(D)J recombination analysis.* The international ImMunoGeneTics information system (IMGT®;) has developed several tools for the in-depth analysis of V(D)J recombinations [8–12]. Many software focuses on V(D)J segmentation, identifying the V, D, and J regions in a sequence. The available V(D)J segmenters perform sequence alignments against full germline databases (JoinSolver [13], V-QUEST [9], HighV-QUEST [11]), possibly with some alignment heuristic ([14], IgBlast [15]), models such as hidden Markov models (HMMs) (iHMMune-align [16], SoDA2 [17]), or maximum-likelihood-based techniques (VDJSolver [18]). A short benchmark of some of these tools has been published [19], but there is the need for more complete and independent evaluation.

*V(D)J analysis of high-throughput sequencing data.* Since 2009, several studies have investigated V(D)J repertoires with high-throughput sequencing, in animals [20–22] and humans, to explore repertoire diversity [4, 14, 23] or in leukemia patients at follow-up [24–28].

Several of those studies used 454 pyrosequencers, which produce long reads but with a lower throughput than some other sequencers. Recently, the study [29] estimated clonal diversity with a pipeline involving IMGT/HighV-QUEST [11], gathering into a “IMGT clonotype (AA)” sequences following a unique V(D)J rearrangement and a unique junction sequence.

Studies that have taken advantage of the higher throughputs available with some Illumina sequencers, such as [4, 30, 31], had to deal with incomplete short reads that did not contain the whole recombination. Several short reads had to be assembled to obtain longer reads covering the whole recombination, requiring that the reads were sufficiently redundant. One recent study that used Illumina sequencing [26] focused on leukemia follow-up on the human immunoglobulin heavy chain. The study [26] accommodated the short reads by sequencing 115 bp from J to V and then 95 bp inside the V region. It is unclear whether such a strategy can be extended to all Igs or TRs. Moreover, these researchers did not provide any software. Wu *et al* focused on T cells to assess the minimal residual disease in leukemia patients, using an Illumina Hi-seq [32].

Advances in high-throughput sequencing will allow the detection of clones at lower concentrations than is possible with conventional techniques in the study of V(D)J repertoires. More importantly, it will allow multiclone follow-up and the detection of emerging subclones at diagnostic concentrations far below that of the main clone identified at diagnosis, as well as full repertoire analysis [33–35]. However, these advances in “repertoire sequencing” (Rep-Seq) make the development of algorithms and software that can accommodate gigabytes of data imperative [36]. The need for dedicated software is all the more necessary because *standard HTS read mapping tools are useless in this context*. They cannot deal with reads containing recombinations, somatic mutations, or large insertions, and therefore a large amount of data — the most useful! — is lost. Finally, the results expected of such an analysis are not the raw V(D)J segmentations of millions of reads; these sequences must be clustered for clone quantification. Again, *generic clustering tools cannot be used*, because sequences with very small differences can be derived from different clones, especially if these differences occur in N-diversity regions.

A solution is to cluster sequences taking advantage of the V(D)J segmentation. On immunoglobulin heavy chains, Chen *et al* proposed a clustering based on the results of iHMMune-align, implemented in the ClonalRelate software [37]. The clustering is based on a Levenshtein distance between CDR3 sequences that further takes into account the VJ assignation produced by iHMMune-align. The complete method has a quadratic time complexity in the input size. In another study, Laserson *et al* followed the dynamics of the immune response after vaccination, by partitioning the reads on the VJ recombinations (obtained with IMGT/V-QUEST), and by using a sequence-based clustering [38].

*Our contribution.* The tools cited above were primarily designed to study a few V(D)J sequences, and some of them take several hours to process millions of reads. We argue that full V(D)J segmentation on these quantities of reads is unnecessary, and that a better strategy for clonality studies is to first cluster the reads derived from the same clone before the time-consuming V(D)J segmentation.

Therefore, we propose a two-stage strategy. We first use an *ultra-fast window prediction*, where a heuristic analysis outputs a *window* overlapping the third complementarity-determining region (CDR3) with the V(D)J junction. We then produce *a clustering of the clones*, based on the similarity of their windows, and then compute a representative sequence for each clone. This sequence can be further processed, possibly with existing analysis software, to obtain its full V(D)J segmentation and other noteworthy information.

This strategy is implemented in an open-source software called Vidjil. Not computing the complete segmentation on each read allows huge time gains. Vidjil processes datasets with 100,000 reads in less than 1 minute on a laptop computer, including the *de novo* quantification of all the main clones. We also show that the predicted windows are specific enough for individual VJ recombinations to be safely clustered. They ensure a high-quality *multiclonal* analysis: We provide evidence for this quality on TR *γ* chains. We further test simulated data with additional mutations. Indeed, extracting such windows corresponds to what is done with conventional PCR primers specifically designed for one recombination. The method is independent of the number of reads, but the more reads that are sequenced, the lower the detection threshold will be.

Note also that the read length from a high-throughput sequencer with sufficient throughput for studying V(D)J diversity does not always cover the full V(D)J rearrangement (more than 400 bp). This problem might be circumvented by randomly fragmenting full-length DNA segments. Our method allows us to analyze randomly fragmented PCR products by focusing on windows rather than on the full read length.

## Methods

### Dataset preparation and sequencing

Bone-marrow samples taken from a patient at diagnosis and after treatment were obtained from the tissue bank “Tumorothèque du Centre de Référence Régional en Cancérologie de Lille (CRRC)” which certified cell cryopreservation quality. Approval for this study was obtained from the Institutional Review Board of CHRU of Lille (CSTMT093) and was in accordance with the Declaration of Helsinki regarding the informed consent of patients. A written informed consent was obtained from the patient.

#### DNA extraction and PCR

We sequenced the bone-marrow samples taken from a patient with B-cell acute lymphoblastic leukemia (B-ALL) showing a TR *γ* rearrangement. The samples were taken at diagnosis and at three different points during the therapeutic follow-up: Fu-1 (35 days), Fu-2 (122 days) and Fu-4 (207 days). Mononuclear cells were isolated from the bone marrow with a Ficoll system, and the genomic DNA was extracted from the lymphoblastic cells with the QIAamp®; Mini Kit. DNA was quantified with the NanoDrop 2000 system®;. We also constructed a dilution scale, starting with the sample taken at diagnosis and serially diluting it 10-fold five times. The PCR used was based on the Biomed-2 guidelines [3]. The IgH, Ig *κ*, and TR *γ* and *δ* recombinations were explored with multiplex PCR (but not the Ig *λ* or TR *α* and *β* recombinations). Because the TR *γ* PCR Vg1-10 was positive at diagnosis, we used the primer set {Vg1, Vg10, J1J2, JP1/2} for this study (Vg1 5’-GGAAGGCCCCACAGCRTCTT-3’, Vg10 5’-AGCATGGGTAAGACAAGCAA-3’, J1J2 5’-GTGTTGTTCCACTGCCAAAGAG-3’, JP1/2 5’-TTACCAGGCGAAGTTACTATGAGC-3’). 500 ng of DNA was used for the amplification of each target in a 96-well GeneAmp®; PCR System 9700 thermocycler controlled by agarose gel electrophoresis. The PCR products ranged in size from 100 bp to 390 bp.

#### Library preparation

The amplicons were first purified with Qiagen PCR MinElute. We then applied the *Amplicon Concatenation Protocol 03/2012* from Life Technologies included with the SOLiD Fragment Library Construction Kit. We end-repaired 300 ng of each amplicon, and then purified them with the SOLiD Library Column Purification Kit. The amplicons were then ligated with T4 ligase and purified with the SOLiD Library Column Purification Kit. The concatenated amplicons (100 ng) were then sonicated with the Covaris system (six cycles, 10% duty cycles, intensity 5, 100 cycles per burst, time 60 s). The fragmented DNA was then processed with the Ion Xpress Plus gDNA and Amplicon Library (01/31/2012), with slight modifications. The SizeSelect Gel (from Life Technologies) was cut at 330 bp and the amplification step was performed with eight cycles. Independent samples were pooled in different amounts to achieve different sensitivities and then processed with PCR on the OneTouch system from Life Technologies. The libraries were sequenced on a Ion Personal Genome Machine (PGM) system with 200-bp kit chemistry.

#### Primary analysis

The raw Ion Torrent flow was transformed to demultiplexed sequences with the Torrent Server from Life Technologies. As PCR Biomed-2 PCR fragments were concatenated by ligation, each sequence was then split into subfragments based on the identification of a known multiplex PCR primer.

### Algorithm overview

To quantify the clonotype abundances starting from a set of reads, the method proceeds through the following two stages:

- the ultrafast prediction of short zones called *w*-windows, which are regions of length *w* overlapping the third complementarity-determining region (CDR3); this prediction is based on substrings (“*k*-words”);
- the identification and clusterization of the clones (relying solely on these *w*-windows), followed by a refined V(D)J segmentation on a representative read inside each clone.

Note that the “sequence assignment” of [14] also used a step based on substrings. However, in that study, the authors eventually computed a full alignment of each gene to the corresponding germline database.

### Ultrafast CDR3 prediction

The purpose of this heuristic analysis is to extract from a read a sequence of length *w*, called the *w-window*, that overlaps the actual CDR3. Our goal is to center the *w*-window as much as possible on the junction region, predicting a window that also contains the 3’ extremity of the V region and the 5’ extremity of the J region.

This analysis is performed in two steps. The first consists of indexing the germline V and J gene databases, and the second is performed on each read and extracts the *w*-window using the information stored in the index. This analysis is very fast and scalable, because no alignment with germline sequences is required.

#### Indexing step

This index is built once at runtime. It could be precomputed and loaded from disk when necessary. Because the germline databases are very small (a few hundred thousand base pairs, at most), it is not difficult to recompute them, and takes only a few seconds.

The index is built on subsequences of length *k*, called “*k*-words”. Every *k*-word from the germline genes is indexed with a specific label: either *V* (or *J*), when the *k*-word is unique to the V (or J) germline (possibly occurring in distinct sequences from the same germline), or *ambiguous* when the *k*-word is common to both V and J germline genes. The value of *k* is chosen so that such ambiguous words are very rare; by default, *k* is between 10 and 13, depending on the germline. For these small values of *k*, the index can be stored as a flat table of size 4^*k*^. Therefore, the index creation runs in time *O*(*r*+4^*k*^), where *r* is the total size of the germline database. For larger values of *k*, the index is stored as a hash table.

#### Prediction step

**Figure 2:**
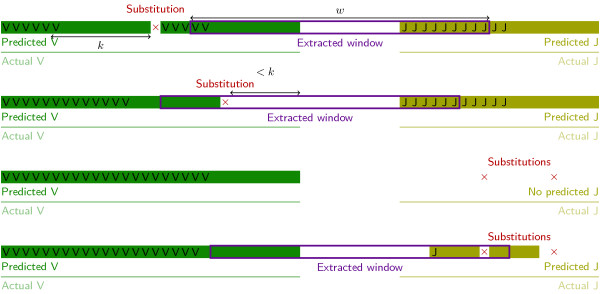
**Heuristic finding a** ***w*** **-window on the forward strand from a scan of** ***k*** **-words in VJ recombinations.** Detection on the reverse strand is done in a similar way, and detection in VDJ recombinations is also based on the V and J genes. The labels *V* and *J* indicate the beginning of matching *k*-words in the index. (Top). The window is correctly centered on the N region (which is between the actual V and the actual J regions). There is one mutation (or sequencing error), denoted by ×, far from the 3’ end of the V region. (Upper middle). A mutation or an error in the *k* rightmost base pairs from the V region leads to a small error in the *w*-window prediction. However, the end of the V region is predicted with an error that is less than or equal to *k*. Because we use large values of *w*, parts of the V and J regions are still contained within the extracted *w*-window. (Lower middle). When there are too many errors compared with the size of the germline gene, the heuristic is unable to predict a *w*-window. This may happen particularly with the J gene, which is shorter than the V gene. For this to occur, mutations must be separated from each other by less than *k* bp. (Bottom). Spaced seeds improve the sensitivity of the heuristic. The spaced 10-word #####-##### leads to the recognition of *k*-words as soon as the mutations are separated by at least *k*/2 bp.

During the second step, each read is considered separately (see Figure 2). We start with the first *k*-word from the read and using the index, we retrieve the value corresponding to that *k*-word and to its reverse complement. We do so for each *k*-word in the read, determining whether the *k*-word is in the V germline, in the J germline, in both, or in neither of them, and on which strand.

At this point, we discard any reads that show an ambiguity, namely reads containing many *k*-words from forward and reverse strands, or reads whose *k*-words are on the forward strand but where *Vk*-words appear after *Jk*-words (and conversely for the reverse strand). To work properly, this rule requires that the V and J germline genes do not share any *k*-words. Hence this constraints the choice of *k*. We must also discard reads for which we have insufficient information: reads that do not have *k*-words found in both the V and J germline genes (Figure 2, lower middle).

Finally, the *w*-window must lie between the last *Vk*-word and the first *Jk*-word (Figure 2, top and middle). Therefore, we extract a *w*-length region centered on that position. The length of the extracted region is a parameter that can be modified by the user. It is set at 40 by default for VJ recombinations. Altogether, the *w*-window prediction step extracts a window in a time that is proportional to the size of the read.

#### Spaced seeds

A further optimization strategy involves using spaced *k*-words, which improve the sensitivity for a fixed specificity [39]. For example, in the spaced 10-word #####-#####, the dash corresponds to a don’t-care symbol. When extracting a subsequence of length *k*+1=11, the middle letter is ignored to form a sequence of length *k*=10. This spaced 10-word minimizes the prediction error in the center of the window when there is one substitution (Figure 2, bottom).

### Read clusterization using *w*-windows

#### Clonal *windows* clusterization

The prediction step extracts one *w*-window per read, at most. If there is no sequencing error, all the extracted *w*-windows for the same clone are strictly identical (Figure 2, top). However, they may not be exactly centered on the actual V(D)J recombination if there are some variants compared with the germline database.

The extracted *w*-windows are then sorted and counted. The relative abundance of each clonotype is then estimated using the number of reads with the same *w*-window. The most abundant clones are kept for detailed analysis.

#### Additional clusterization

Sequencing errors may lead to different *w*-windows that should be gathered in a unique clone (Figure 2, top and upper middle). We recommend the manual inspection of the most abundant clones, because it is then possible to specify in the software pairs of similar windows that must be gathered for analysis. We also provide, as an option, automatic clustering, where two junctions are considered similar if their edit distance is bounded by some parameters.

#### Computation of representative sequences

The previous steps identified clones as a set of reads sharing the same *w*-window (or similar *w*-windows if additional clusterization has been used). We then select one representative sequence per clone, and thus compute only one V(D)J segmentation per clone. Because this segmentation will be used to label all the reads of the clone, we must select the representative sequence carefully.

To do so, we start by counting all the *k*-mers of reads belonging to a given clone. This is done using a hash table. We call any subsequence of a read whose *k*-mers are present above a relative threshold (*e.g.* 50 % of the number of sequences in the clone) a *representative region*. Reads are considered one by one, and we output the longest representative region among all the clone’s reads. Obviously, this representative region must overlap the *w*-window that has been formerly detected. This computation is linear time in the number of nucleotides in the sequences belonging to that clone. Therefore the bigger the clone, the more time it will take. Computing this region further allows us to check the consistency of the reads assigned to the same clone.

#### Refined V(D)J segmentation

The representative sequence identified for each clone can be segmented into V(D)J regions using any available segmenter [9, 11, 13, 15–18]. To give a first hint on the V(D)J segmentation, we also implemented a basic segmenter using dynamic programming against a database of germline genes. This segmentation runs, for each representative sequence of length *ℓ*, in *O*(*ℓr*) time, where *r* is the size of the database of the germline gene. This segmentation is not at the core of the read clusterization and is provided only for convenience.

### Time complexity

The prediction of junctions is in linear time, so the whole algorithm is very scalable because there are often very few *w*-windows of interest that are left to the most time consuming steps – the computation of the representative sequence and the full V(D)J segmentation.

### Software

The algorithms were implemented in C++ in an open-source software called Vidjil. The software can be downloaded from http://www.vidjil.org. The software takes as the input a set of reads and a database of germline genes. In all our experiments, we used the IMGT/GENE-DB database [40] downloaded from http://www.imgt.org.

Vidjil outputs the list of *w*-windows detected and the most frequent clones. As explained above, the detection of *w*-windows is based on spaced *k*-mers extracted with seeds. By default the seed used for TR *γ* germline is #####-##### of weight 10. On this germline, there is no spaced *k*-word with this seed common to both V and J genes: There is thus very few chances to falsely discard reads. Depending on the receptor, there can be more overlap between *k*-mers of V and J genes. In this case, or when there are more mutations or errors in the dataset, longer seeds should be used to improve the ratio of *w*-windows detected. By default, Vidjil uses a seed of weight 12 for TR *β* and IgH and a seed of weight 13 for TR *α*. The user can also specify his own seed, or any value of *k* for a contiguous seed.

Vidjil will output the 20 most abundant clones with their representative sequence and their refined V(D)J segmentation. It will not process clones with less than 10 reads. These parameters can be changed by the user. The user can also follow other clones, even if they are not among the most frequent ones, by specifying their *w*-window.

The user can define the maximum number of substitutions, indels, and homopolymer errors that can be accepted between two similar windows. By default, we tolerate none. These parameters should be set depending on the sequencer used and should be very conservative to prevent any false clustering of different clones.

Table 1 compares the running times of Vidjil and other programs. Vidjil is very fast and further produces clusters whereas other methods output information at the read level. Note that it is also possible to launch the programs on a set of unique reads (between 61% and 81% of the reads in our samples). In this case, the running times of the three programs stay in the same proportions, Vidjil still being the fastest.

**Table 1:** **Running times of the different programs on a test set of 100,000 reads**

	Vidjil	HighV-QUEST	IgBlast
time	18s	1 hour	3m 50s
availability	standalone	website	website, standalone

Vidjil (version 2013.10) and IgBlast (version 1.2.0) were launched on a laptop with a 2 GHz processor (1 core used) and 8 GB of memory. IMGT/HighV-QUEST (version 3.2.31) was launched on the IMGT web server. The web server of IMGT/HighV-QUEST is limited to 500,000 sequences.

## Results

### Dataset

The bone-marrow samples were obtained from a patient with B-ALL showing a TR *γ* rearrangement. The samples were taken at diagnosis (Diag) and at three follow-up points (Fu-1, Fu-2, and Fu-4, collected at 35, 122, and 207 days after diagnosis, respectively). A standard curve was established from serial dilutions of the diagnosis samples in a peripheral blood lymphocyte (PBL) solution mixed from five healthy donors, producing samples Scale- 10^-2^, Scale- 10^-3^, Scale- 10^-4^, and Scale- 10^-5^.

Those eight samples were sequenced as described in methods and can be accessed at http://www.vidjil.org/data. In Additional file 1: Table S1, we provide statistics on these samples. We recall that on the TR *γ* chain, recombinations are in the VJ form. The number of reads differed for each dataset because the same coverage was not required for each of them for validation purpose. For instance, we need better coverage for the 10^-5^ dilution than for the diagnosis sample, where the majority of the sequences are expected to correspond to one clone. The DNA fragments were previously concatenated and randomly fragmented. Note that the goal of this sequencing is to assess the speed and robustness of Vidjil and not to achieve the lowest possible detection threshold, which depends on the number of reads and the sequencing protocol used.

### Evaluation of the window prediction

The window prediction phase is a heuristic that does not rely on dynamic programming and may therefore be less accurate than a more time-consuming algorithm. We assess the accuracy of the Vidjil heuristic on our datasets by comparing the location of the detected *w*-window with the ones predicted by IMGT/HighV-QUEST [11] and IgBlast [15]. Our goal is not to assess the IMGT/HighV-QUEST and IgBlast software, but to verify that the Vidjil’s heuristic is sufficiently accurate. Even if ClonalRelate [37] is of interest we could not compare to it since it builds upon results provided by iHMMuneAlign, that is specifically dedicated to immunoglobulin heavy chain analysis.

We selected two datasets for this comparison: Diag, which contains high redundancy and a lower number of reads; and Scale- 10^-5^, which is supposed to have much greater diversity.

IgBlast (version 1.2.0) was launched in its stand-alone version. We launched IgBlast using the TR *γ* germline database downloaded from IMGT/GENE-DB [40]. The other parameters were left at the default settings. Only the “top segmentation” returned by IgBlast was kept, consisting of the top V and J gene matches. IMGT/HighV-QUEST was launched by specifying the organism (Human) and the locus (TR *γ*); by specifying that the sequences originate from a single individual; and by allowing indels. The other parameters were left at the default settings.

What was compared among these three tools was the position of the center of the window. IMGT/HighV-QUEST and IgBlast do not give this position, but it can be computed easily from the 3’ position of the V region and the 5’ position of the J region, which are given.

**Figure 3:**
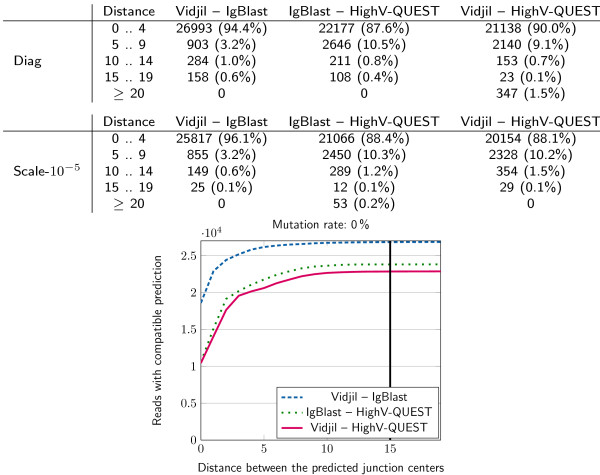
**Comparison of the predictions of the center of the window made with IgBlast, IMGT/HighV-QUEST, and the heuristic of Vidjil, on the 100,000 first reads of a diagnosis sample (Diag, top) of a patient with ALL and on a dilution (Scale- 10** ^**-5**^ **, lower table and graph).** For each pair of programs, the number shows the distance between the predictions of the center of the window overlapping the CDR3. These values show that Vidjil successfully predicted the center of the windows. Note that the two other tools provided much more information, with alignments to the germline databases, and in the case of IMGT/HighV-QUEST, further analysis of the junction sequence.

**Figure 4:**
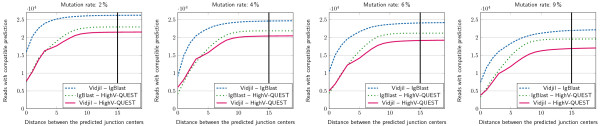
**Comparison of the predictions of the center of the window overlapping the CDR3 made with IgBlast, IMGT/HighV-QUEST, and the heuristic of Vidjil, on the 100,000 first reads on a dilution (Scale- 10** ^**-5**^ **).** Additional mutations (2%, 4%, 6%, 9%) are added by simulation. Even with 6% mutations, the heuristic of Vidjil locates almost all the junction centers within 15 bp of the center found by other programs (99.4% of the commonly segmented reads).

- The results for the actually sequenced dataset (see Figure 3) show that the center of the window predicted by Vidjil differed from those predicted by IMGT/HighV-QUEST and IgBlast by less than 10 positions in more than 97% of cases, and by less than 15 positions in about 99% of cases. Vidjil shows a greater concordance with IgBlast than with IMGT/HighV-QUEST. The reason may be that IMGT/HighV-QUEST is conceived for longer sequences. Our dataset may contain short sequences that Vidjil is also able to process.
- As B cells are subject to somatic hypermutations, it is more difficult to segment their sequences. We can assess the robustness of the method against mutations by adding substitutions to our sequenced dataset. In the literature, estimates of the rate of sequence substitutions arising from somatic hypermutation are around 2% [41, 42]. Arnaout *et al* empirically estimated hypermutations in humans to be about 5% to 8% [14]. We generated datasets with 2%, 4%, 6% and 9% random substitutions along each read. Those datasets can be accessed at http://www.vidjil.org/data. Note that those substitutions are added to the errors that may have been produced by the sequencers. The results for the mutated datasets (see Figure 4) show that on reads with 6% additional mutations, the center of the window predicted by Vidjil differed from that predicted by the other programs by less than 15 positions in about 99.4% of the cases. Vidjil shows again a greater concordance with IgBlast than with IMGT/HighV-QUEST.

For VJ recombinations, such as in TR *γ*, a positional inaccuracy of up to 14 bp is not a problem because we are using 40 bp *w*-windows. The predicted window will still contain the N-diversity region, allowing the correct identification of the clones. However, a window lying only in a V region or a J region would be problematic. In that case, the window would be overrepresented and would lead to the detection of false clones. For VDJ recombinations, Vidjil predicts 60 bp windows to ensure that the complete N-diversity regions are included in the detected *w*-window.

Therefore, the window prediction accuracy of Vidjil is such that just a small fraction of sequences may have a wrong window. It is noteworthy that when IMGT/HighV-QUEST and IgBlast are compared, the difference between them is similar to the difference between them and the prediction heuristic of Vidjil.

### Evaluation of Vidjil sensitivity

Note that the detection threshold depends directly on the number of reads actually sequenced. A recent study, using a higher-throughput sequencer, reported a detection threshold of 10^-6^[27, 28]. Our goal is not to achieve the lowest possible threshold, but to show that Vidjil can correctly estimate the relative concentrations of the clones.

**Figure 5:**
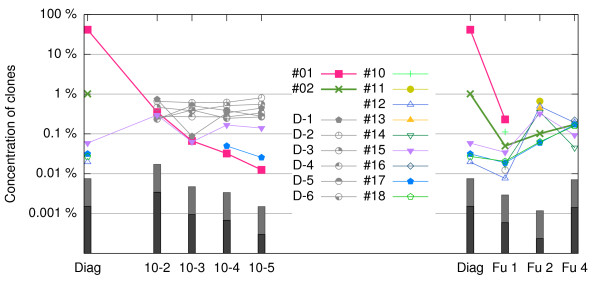
**Evolution of the main TR** ***γ*** **clones from a patient with ALL, starting at diagnosis and diluted to decreasing concentrations of 10** ^**-2**^ **, 10** ^**-3**^ **, 10** ^**-4**^ **, and 10** ^**-5**^ **(left part of the plot); and when the patient is followed at three time points (Fu1, Fu2, and Fu4, right part).** Clones #01 and #02 are the two most abundant clones detected at diagnosis, and the other clones are among the five most abundant clones, for at least one sample. Clones D-1 to D-6 are found in at least two of the dilutions, but are never found in any sample that is not a dilution. The black and gray boxes below each point indicate the maximum resolution, depending on the number of reads of each sample (black: 1 read, absolute detection threshold; gray: 5 reads, detection threshold to consider that the clone is significant). A sequencing with more reads will improve these thresholds.

Figure 5 shows the relative concentrations of the most abundant clones in each sample. We launched Vidjil on each of those eight samples, retrieving the five most abundant *w*-windows in each sample, and manually reviewed those windows to cluster them into clones. The plots represent the concentration ratios of those clones in any of the samples.

**Figure 6:**
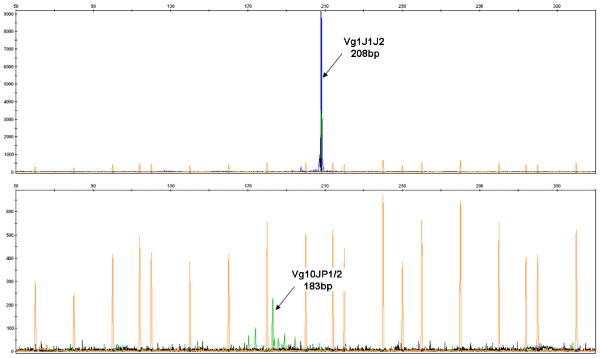
**Fluorescent PCR of the diagnosis sample (Diag) of a patient with ALL.** (Top) A 208-bp peak is detected with multiplex PCR of TR *γ* Vg1-10, corresponding to the main #01 clone TRGV5*01 -5/CC/0 TRGJ1*02. (Bottom) A Vg10-JP1/2-specific PCR shows a 183 bp peak, similar in size to clone #02 (185 bp) detected by Vidjil on the high-throughput sequencing data.

*Clones at diagnosis.* Table 2 details the two most abundant clones at diagnosis (Diag). The most abundant clone, labeled #01, is the one with recombination TRGV5*01 -5/CC/0 TRGJ1*02. This clone is exactly the one that was followed in this patient with standard techniques, and was observed by fluorescent multiplex PCR analysis (Figure 6, top). As expected, this clone is most abundant.

**Table 2:** **Two most abundant TR** ***γ*** **clones detected in 100,000 sequences from diagnosis sample (Diag) of a patient with ALL**

				Vidjil	IMGT/V-QUEST	IgBlast
Clone #01	TRGV5*01	-5/CC/0	TRGJ1*02	9 204 reads	7 376 reads	11 319 reads
	... GTGCCACCTGGG	CC	TTATTATAAGAA...	(31.9%)	(42.1%)	(36.3%)
Clone #02	TRGV10*02	-5/AGAC/ -3	TRGJP1*01	253 reads	175 reads	353 reads
	... TGTGCTGCGTGG	AGAC	CCACTGGTTGGT...	(0.88%)	(0.80%)	(1.1%)

In this sample, 28 809 reads have been segmented by Vidjil, 29 039 by IMGT/HighV-QUEST (and 21 876 when taking into account IMGT/JunctionAnalysis results) and 31 147 by IgBlast. For each method, the number of associated reads is given. The VJ segmentation proposed by Vidjil was manually checked against the analysis provided by IMGT/V-QUEST and IgBlast. Clone #01 has the recombination TRGV5*01 -5/CC/0 TRGJ1*02, which means that the V gene is TRGV5*01, according to the IMGT nomenclature, and its last five nucleotides have been deleted. The N-diversity region is composed of two inserted Cs, and the J gene is TRGJ1*02, which has no deletion.

The second most abundant clone (#02), at approximately 1 %, was identified as TRGV10*02 -5/AGAC/-3 TRGJP1*01. It was not initially detected at diagnosis with standard procedures, and was consequently not followed in this patient. A further fluorescent simplex PCR analysis with specific primers showed several peaks, including a major peak at 183 bp (Figure 6, bottom), similar in size to that of clone #02 detected with Vidjil (182 bp).

The Table 2 also shows that the predictions made by Vidjil are coherent with the ones made by IMGT/HighV-QUEST or IgBlast. Note that Vidjil process slightly less sequences that IgBlast: The main reason is that IgBlast can provide J gene affectation with very few nucleotides in the J gene, while Vidjil needs at least *k* conserved nucleotides. Concerning quantification estimation, IMGT/HighV-QUEST and IgBlast do not provide the raw result of clone quantification but it can be easily computed by gathering sequences with the same junction. We emphasize on the fact that IMGT/HighV-QUEST works better when processing longer sequences (*e.g.* reads from 454 sequencer). The two main clones are found at the same level by the three softwares even if the number of segmented sequences differ among them. Vidjil’s quick heuristic does not prevent it from correctly clustering reads coming from a same clone.

*Dilution clones.* The dilution samples (samples Scale- 10^-2^ to Scale- 10^-5^) are composed from 99% to 99.999% of the same PBL solution. It is thus meaningful that in these samples, the concentration ratios of the most abundant clones remain remarkably stable throughout the dilutions. These clones should be specific to the PBL, and not to the patient.

Generally, Vidjil can distinguish clones that are different with great accuracy by focusing on the *w*-windows. When there is no further window clusterization, the reads reported to belong to the same clone share exactly the same *w*-window. However, some clones were found at similar concentration ratios in both the PBL and patient samples, such as clone #15, identified as TRGV10*02 -4//0 TRGJP1*01. This clone could be either what was called a “public sequence” by [43], that is a recombination being shared by different people or a random recombination. There may be also some PCR artifacts. Note that TR *γ* does not show great diversity (18 distinct V genes and six distinct J genes according to the IMGT germline databases) and this clone has no inserted N-diversity region.

*Follow-up points.* The concentration of clone #01, measured with standard procedures (compared with the total number of cells), was 94% for Diag, 0.5% for Fu-1, 0.05% for Fu-2, and ≤ 0.5% for Fu-4. The ratios of the rearranged TR *γ* sequences show a similar evolution, even if there is some bias, which could be corrected with a better calibration of the wet-lab protocol.

## Discussion

High-throughput sequencers will eventually raise the detection threshold, as already reported by several studies. They will also provide full insight into the whole population of lymphocytes, with *multiclonal* analyses of such populations. We believe that these analyses will bring a better understanding of lymphoid malignancies, and more generally, of immunology. However, they require specifically adapted mapping and clustering tools.

We have proposed new algorithms designed for data from high-throughput sequencers. We have not focused on the analysis of individual reads, but have instead based the method on the ultrafast detection of windows containing the actual recombination junctions. As a consequence, the Vidjil program can process large datasets in a few minutes, outperforming other methods that are more adapted to the full analysis of individual sequences. The method applies to any number of reads: The more reads that are sequenced, the lower the detection threshold will be.

Our window definition, used to define a clone, differs from what can be found elsewhere in the literature [4, 29, 38] in that we do not rely on the VJ gene names and we focus on the DNA sequence at the junction (while some use the amino acids) without allowing any mismatch by default (while others allow mismatches). Hence we think that our definition appears to be more stringent. Our belief is that we should avoid putting together sequences that should not be together. On the other hand our definition may split sequences that should be together but if one wants to allow more errors the sequences can be further clustered.

Our results for sequenced and artificially mutated data show that the window prediction, clusterization, and representative sequence selection are accurate enough for clone tracking. This was confirmed both for raw TR *γ* data and for mutated data, showing that the method can gather clones with a dissimilarity of up to 6%, arising from random mutations mimicking hypermutations. We tested Vidjil on TR *γ* which is less diverse than other loci. Hence if Vidjil had a lack of reliability, we would have been able to identify it. On the contrary we observed that the results are consistent both with conventional methods and with software focusing on a more in-depth analysis.

As the Vidjil heuristic is fast and reliable, it could be used as a pre-processing for other programs. Indeed the purpose of Vidjil is not to provide detailed information on a given sequence. Several software are designed for that purpose: For example, one may launch IMGT/V-QUEST, IgBlast, or, for IgH clones, iHMMune-align for an in-depth analysis of the clones identified by Vidjil. Starting from Vidjil strict definition of clones, one could also use software such as ClonalRelate [37] to further gather these clones and to study their relationship.

Note that all the ratios were computed by taking the number of segmented reads as a reference, which ideally corresponds to the number of rearranged T or B cells in the studied system. This differs from the proportion of the total cells used in current protocols, which also include other mononucleic cells, such as precursor cells. The inclusion of a standard of known concentration could be used to calibrate these different ratios.

## Conclusions

When used to monitor minimal residual disease, Vidjil can successfully follow the variations in the main clone. It also identifies other stable clones that could be investigated to determine whether they are pathological or physiological. Given samples taken at different times, the method enables to track the evolution of a population of clones and to check the emergence of new clones. The method could also be used for other immunological studies to quantify more precisely the adaptive immune response and the long-term immunological memory.

## Electronic supplementary material

Additional file 1: **Additional information regarding sequencing data.** (PDF 80 KB)
