# Supplementary material for: Fast multiclonal clusterization of V(D)J recombinations from high-throughput sequencing
Source: BMC Genomics. 2014 May 28;15(1):409. doi: 10.1186/1471-2164-15-409 (PMC4070559; doi:10.1186/1471-2164-15-409)
Supplement: Supplementary file 1 — Additional file 1: Additional information regarding sequencing data. (PDF 80 KB) [file 12864_2013_6130_MOESM1_ESM.pdf]

# Supplementary data for "Fast multiclonal clusterization of V(D)J recombinations from high-throughput sequencing"

October 9, 2013

| | reads | 40-windows detected | | unique 40-windows | clones with $\geq$ 100 reads |
|---|---|---|---|---|---|
| Diagnosis (Diag) | 191 095 | 62 461 | (32.7 %) | 13 074 (20.9%) | 18 |
| Scale-$10^{-2}$ | 85 903 | 27 734 | (32.3 %) | 12 430 (44.8%) | 3 |
| Scale-$10^{-3}$ | 292 535 | 100 990 | (34.5 %) | 34 056 (33.7%) | 17 |
| Scale-$10^{-4}$ | 437 490 | 142 230 | (32.5 %) | 44 499 (31.3%) | 32 |
| Scale-$10^{-5}$ | 969 026 | 315 011 | (32.5 %) | 80 548 (25.6%) | 302 |
| Follow-up 1 (Fu-1, 35 days) | 624 078 | 161 458 | (25.9 %) | 66 435 (41.1%) | 13 |
| Follow-up 2 (Fu-2, 122 days) | 1 360 656 | 406 606 | (29.9 %) | 95 862 (23.6%) | 807 |
| Follow-up 4 (Fu-4, 207 days) | 375 510 | 66 361 | (17.7 %) | 29 633 (44.6%) | 6 |

Table 1: Statistics on the eight samples sequenced for a patient with ALL and on the results of Vidjil. On each dataset, starting from some number of reads, Vidjil detects 40-windows overlapping the CDR3. The count of unique 40-windows is smaller as clones gather several reads. The last columns shows the number of clones with at least 100 reads. All percentages are computed with respect to the number of reads of each dataset.
